# Multicenter Testing of the Rapid Quantification of Radical Oxygen Species in Cerebrospinal Fluid to Diagnose Bacterial Meningitis

**DOI:** 10.1371/journal.pone.0128286

**Published:** 2015-05-26

**Authors:** Anne-Claire Lukaszewicz, Valérie Faivre, Hélène Bout, Etienne Gayat, Tina Lagergren, Charles Damoisel, Damien Bresson, Catherine Paugam, Jean Mantz, Didier Payen

**Affiliations:** 1 Department of Anesthesiology and Critical Care, Lariboisière Hospital, Univ Paris Diderot, Sorbonne Paris Cité, Paris, France; 2 Department of Anesthesiology and Critical Care, Beaujon Hospital, Univ Paris Diderot, Sorbonne Paris Cité, Paris, France; 3 Department of Neurosurgery, Lariboisière Hospital, Univ Paris Diderot, Sorbonne Paris Cité, Paris, France; Anhui Medical University, CHINA

## Abstract

**Purpose:**

Meningitis is a serious concern after traumatic brain injury (TBI) or neurosurgery. This study tested the level of reactive oxygen species (ROS) in cerebrospinal fluid (CSF) to diagnose meningitis in febrile patients several days after trauma or surgery.

**Methods:**

Febrile patients (temperature > 38°C) after TBI or neurosurgery were included prospectively. ROS were measured in CSF within 4 hours after sampling using luminescence in the basal state and after cell stimulation with phorbol 12-myristate 13-acetate (PMA). The study was conducted in a single-center cohort 1 (n = 54, training cohort) and then in a multicenter cohort 2 (n = 136, testing cohort) in the Intensive Care and Neurosurgery departments of two teaching hospitals. The performance of the ROS test was compared with classical CSF criteria, and a diagnostic decision for meningitis was made by two blinded experts.

**Results:**

The production of ROS was higher in the CSF of meningitis patients than in non-infected CSF, both in the basal state and after PMA stimulation. In cohort 1, ROS production was associated with a diagnosis of meningitis with an AUC of 0.814 (95% confidence interval (CI) [0.684–0.820]) for steady-state and 0.818 (95% CI [0.655–0.821]) for PMA-activated conditions. The best threshold value obtained in cohort 1 was tested in cohort 2 and showed high negative predictive values and low negative likelihood ratios of 0.94 and 0.36 in the basal state, respectively, and 0.96 and 0.24 after PMA stimulation, respectively.

**Conclusion:**

The ROS test in CSF appeared suitable for eliminating a diagnosis of bacterial meningitis.

## Introduction

Traumatic brain injuries and neurosurgical procedures (craniotomy, ventriculostomy catheterization and external ventricular drainage (EVD)) confer a high risk of nosocomial meningitis. The incidence varies between 1.4% [1], 0.8–1.5% [2] and 8% [3] with a major impact on outcome. Diagnosing meningeal or intracranial infection is difficult in this context because clinical symptoms are nonspecific and are frequently attenuated by steroid treatment or induced hypothermia. In addition, the classic criteria for cerebral-spinal fluid (CSF) infection, such as pleiocytosis with a high proportion of polymorphonuclear cells (neutrophils; PMNs), low glucose and high protein levels, are difficult to apply in this context after recent bleeding or surgical procedures, especially when antibiotics have been given for prophylaxis or previous infections [4]. When meningitis is suspected, the clinician usually repeats the CSF analysis and finally gives broad spectrum antibiotics until the microbiology results are available (from 48 to 72 hrs) because of the risk in delaying treatment [5]. This attitude may lead to the repetition of CSF testing and potential selection of multiresistant micro-organisms. The availability of a rapid, specific, and sensitive test to diagnose increased CSF inflammation would help therapeutic decision-making.

PMNs are the first line of defense of the innate immune response to bacterial infection and are activated when infection is present. Such activation is essential for many PMN functions, especially crossing the blood brain barrier and releasing radical oxygen species (ROS) [6]. In a recent study, we described a rapid method for assessing the production of ROS in CSF using a luminescence method [7]. The method was adapted to perform a quick diagnosis of CSF bacterial infection and was compared to sterile inflammation in patients with EVD for intracranial hemorrhage.

This study reports the results of a prospective multicenter study testing ROS measurement by luminescence to diagnose nosocomial meningitis. The test was initially applied to a single-center cohort (cohort 1) as a training cohort and prospectively applied to multicenter cohort 2 for validation.

## Patients and Methods

This study was approved by the ethical committee of the Société de Réanimation de Langue Française (IRB00006477, n° 10–071) and conducted according to instructions; patients or their next of kin received written information. Signed informed consent was not required because measurements were conducted with routine CSF samples without further biological collection. Patients from two teaching hospitals were enrolled in the intensive care unit (ICU) or in the neurosurgery department when they had a fever over 38°C in a context of TBI, after neurosurgery or CSF drainage by catheterization (EDV). The daily enrolment time was between 7 AM to 7 PM because ROS measurements should be performed on fresh samples with a delay after sampling of less than 4 hours. The first single-center cohort (cohort 1) was prospectively studied from Feb 2009 to Oct 2010 (54 patients in the Surgical ICU at Lariboisière University Hospital). The multicenter cohort (cohort 2) was prospectively enrolled for validation of the test from Oct 2010 to Mar 2012 (136 patients; Surgical ICUs and Department of Neurosurgery; Lariboisière University Hospital and Beaujon University Hospital). The physicians in charge of the patients were not aware of the investigation and ignored the results of ROS measurements when managing their patients.

### Clinical and biological data collection

For each patient, the following data were collected: demographic characteristics, type of surgery if any, presence of external CSF drainage, method for CSF sampling (lumbar puncture or catheter drainage), symptoms of inflammation; routine CSF laboratory analysis (cell count/ml, protein g/L, and glucose mmol/L), medical treatments, especially antimicrobial therapy at the time of sampling, and outcome. If present, other concomitant sites of infection were considered.

### Diagnosis of meningitis

In the absence of a consensus for the definition of variables and thresholds for bacterial meningitis in the context of secondary infection after trauma or neurosurgery [8], we asked two blinded experts (one senior neurointensivist (CD) and one senior neurosurgeon (DB)) to classify patients for meningitis diagnosis after checking medical information, including microbiology results. They applied the adjudication process and came to an agreement in each case. These two experts were not in charge of the protocol.

### Measurements of ROS production by luminometry

ROS production in CSF was evaluated by luminescence intensity (luminometer, AutoLumat *Plus* LB 953; Berthold Technologies, Bad Wildbad, Germany) with luminol (Sigma, Saint Quentin Fallavier, France) measured under basal conditions and after cell stimulation with phorbol 12-myristate 13-acetate (PMA; Sigma) as previously described [7]. To ensure method homogeneity, all samples were centralized in a single laboratory (Anesthesiology Lab; Lariboisière Hospital) and measurements were performed within 4 hours. The obtained signal, expressed in Relative Light Units (RLU), was recorded over 20 min and expressed as the area under the curve (AUC) of luminescence over the 20 min. We duplicated the majority of measurements, depending on the quantity of CSF available: 52% of samples were studied in duplicate for both basal and PMA conditions (250 microliter x2 of CSF required per condition). The potential variability in the reactivity of luminol was avoided by regularly preparing new luminol solutions and repeating tests over several weeks with decreasing concentrations of stable H_2_O_2_ (Chimie Recherche Environnement Evolution, Taverny, France). These tests showed a good reproducibility of the signal.

### Statistical analysis

Values were expressed as median and interquartile range or count and percentage. Groups of patients were compared using the chi-square test for categorical variables and the Student’s t test or Wilcoxon test for continuous variables as appropriate. A diagnosis of meningitis was based on the consensus of two experts. To determine the diagnostic accuracy of the luminescence level measured either under basal conditions (AUC ROS production under basal conditions) or associated with cell function (AUC ROS production under PMA conditions), operating characteristics of both tests were evaluated using receiver operating characteristics curves (ROC) with calculation of the area under the curve (AUC). Confidence intervals (95%) of AUCs were estimated using bootstrap operations [9]. For all analyses, luminescence values were log_natural_-transformed. Sensitivity, specificity, negative predictive value, positive predictive value and likelihood ratios of best cut-off values were calculated for the two measurements of luminescence according to the Youden index (defined as the sum of [sensitivity+specificity-1]). Statistical analyses were performed using R-statistical software (http://www.r-project.org/). A two-sided P value <0.05 was considered statistically significant.

In the sensitivity analysis, the performance of the ROS test was compared to formerly published meningitis criteria including CSF cell count > 100/mm^3^ and positive microbiology culture [5].

## Results

The characteristics of the two cohorts are presented in Table 1. All patients were febrile (>38°C) with a suspicion of meningitis related to the risk of penetration after trauma (25%, delay of 8 days (5–14.75), median (25–75^th^ percentiles)) or major neurosurgery with a leak or in an emergency (38%, delay of 9 days (5–14)), or external ventricular drainage (delay of 9 days (4.5–13)). Six patients were taking hydrocortisone, 5 were taking prednisone and 2 had recently received non-steroidal anti-inflammatory drugs.

**Table 1:** Clinical context and characteristics of the two cohorts.

	Cohort 1 (n = 54)	Cohort 2 (n = 136)
Postop, n (%)	22 (40.7)	51 (38.3)
Trauma, n (%)	12 (22.2)	36 (27.1)
EVD, n (%)	20 (37)	46 (34.6)
Body temperature (°C)	38.8 (38.3 to 39.4)	39 (38.5 to 39.4)
WBC (10^3^/mm^3^)	13.1 (9.6 to 18)	13.6 (10.3 to 18.1)
% of PMN in blood	79.2 (74.6 to 83.8)	80 (75 to 84)
Glycemia (mmol/L)	7.65 (6.4 to 9.75)	7.4 (6.2 to 8.8)
Number of patients on ATB (%)	31 (58)	53 (39)
CSF characteristics		
Cell count (/mm^3^)	30 (8 to 475)	33 (7 to 210)
PMN count (/mm^3^)	74 (26 to 89)	66 (41 to 86)
Lymphocyte count (/mm^3^)	10 (3 to 45)	20 (8 to 44)
RBC (/mm^3^)	6000 (893 to 52000)	2000 (230 to 12700)
W/R	0.75 (0.151 to 8.257)	1.4 (0.17 to 14.935)
CSF glucose (mmol/L)	3.9 (2.45 to 4.6)	3.55 (2.73 to 4.57)
CSF/blood glucose	0.5 (0.4 to 0.7)	0.5 (0.4 to 0.6)
Proteins (g/L)	1 (0.5 to 1.88)	0.8 (0.36 to 1.46)
Positive microbiology n (%)	11 (20.4)	22 (16.2)
Adjudicated diagnosis of meningitis n (%)	14 (25.9)	21 (15.4)

Quantitative parameters are expressed as median values (min to max).

Postop: postoperative, Trauma: traumatic brain injury, EVD: external ventricular drainage, WBC: white blood cells, CSF: cerebrospinal fluid, PMN: polymorphonuclear cells, RBC: red blood cells, W/R: white blood cells/red blood cells, ATB: antibiotics.

### Patients and clinical diagnosis of meningitis in cohort 1

Fifty-four patients were included in cohort 1. Meningitis was diagnosed by the experts in 14 patients from this cohort when CSF was positive for microbes in 11 patients (4 for *Pseudomonas*, 2 for *Staphylococcus*, 2 for enterobacteria, *Enterococcus*, *Acinetobacter* and *Gemella*). A discrepancy between the diagnosis and microbiology findings was observed for 7 patients (13.0%) (see electronic supplement S1 Table). Meningitis was diagnosed despite negative CSF culture (n = 5) because of high PMN counts, low CSF glucose levels and high protein levels in a post-surgical context to avoid the risk of missing the diagnosis. A negative diagnosis was made by the experts despite positive CSF culture (*Staphylococcus epidermidis*, *Staphylococcus aureus and Streptococcus spp*) in two patients because of low CSF cell counts, low protein and high glucose levels in a context of extraventricular drainage. The experts thought that this pattern was more likely due to material colonization than infection. Differences in clinical and bacteriological criteria according to the diagnosis of meningitis are detailed in Table 2.

**Table 2:** Clinical and biological characteristics in cohorts according to the diagnosis of meningitis.

	Cohort 1			Cohort 2		
	No meningitis (n = 40)	Meningitis (n = 14)	p value	No meningitis (n = 115)	Meningitis (n = 21)	p value
Body temperature (°C)	38.7 (38.3 to 39.2)	39.5 (38.4 to 40.3)	0.17	39 (38.5 to 39.2)	39 (38 to 39.5)	0.73
Blood leukocytes (10^3^/mm^3^)	12.3 (9.6 to 15.3)	16.6 (12.9 to 21.3)	0.036	13.6 (10.2 to 18)	13.8 (12.1 to 18.9)	0.43
% of blood PMN	78.4 (72.2 to 82.4)	83.4 (83 to 84.1)	0.07	79 (75 to 83)	87 (80 to 89)	0.0014
Cells in CSF (/mm^3^)	20 (4 to 85)	500 (280 to 1830)	0.00011	20 (5 to 114)	1000 (233 to 3660)	0.012
% of PMN in CSF	68 (8 to 87)	84 (68 to 89)	0.097	57 (28 to 80)	80 (74 to 89)	0.006
% of lymphocytes in CSF	13 (0 to 54)	8 (4 to 22)	0.77	25 (10 to 52)	13 (4 to 22)	0.0049
Red cells in CSF	6000 (1661 to 53000)	820 (20 to 14800)	0.12	2935 (282 to 13750)	600 (230 to 1600)	0.72
W/R in CSF	0.424 (0.066 to 1.731)	60 (3.378 to 1545.946)	<0.0001	0.877 (0.134 to 6.402)	118.182 (8.917 to 504.348)	0.022
CSF glucose (mmol/L)	4.2 (3.2 to 5.2)	2.45 (2.15 to 3.05)	0.013	3.8 (3.12 to 4.7)	1.9 (0.5 to 2.7)	<0.0001
Glycemia (mmol/L)	7.8 (6.4 to 9.8)	7.5 (5.9 to 9.2)	0.82	7.2 (6.1 to 8.67)	8.2 (7.3 to 9.3)	0.16
CSF/blood glucose	0.5 (0.4 to 0.7)	0.3 (0.3 to 0.5)	0.066	0.5 (0.4 to 0.6)	0.2 (0.1 to 0.3)	<0.0001
CSF proteins (g/L)	0.8 (0.5 to 1.3)	2.6 (1.1 to 3.2)	0.0014	0.63 (0.3 to 1.19)	1.6 (1.17 to 3.55)	0.041
Number of patients on ATB (%)	23 (57.5)	8 (61.5)	0.80	45 (39.1)	8 (38.1)	0.93
ROS after PMA stimulation (AUC)	664830 (69398 to 1495020)	9381030 (1098750 to 92859240)	0.00094	1522020 (115410 to 11520060)	63588420 (7887139 to 188649184)	<0.0001
ROS in the basal state (AUC)	16965 (9375 to 41768)	220470 (57195 to 1103415)	0.0006	28050 (13252 to 151470)	877245 (138540 to 3914745)	<0.0001
Log AUC for PMA stimulation	5.82 (4.84 to 6.17)	6.97 (6.04 to 7.97)	0.00094	6.18 (5.06 to 7.06)	7.75 (6.85 to 8.27)	0.00021
Log AUC for basal state	4.23 (3.97 to 4.62)	5.34 (4.76 to 6.04)	0.0006	4.45 (4.12 to 5.18)	5.94 (5.14 to 6.59)	<0.0001

Results are expressed in median value (min to max); statistics were produced using the Wilcoxon test.

WBC: white blood cells, CSF: cerebrospinal fluid, PMN: polymorphonuclear cells, RBC: red blood cells, W/R: white blood cells/red blood cells, ROS: radical oxygen species, AUC: area under the curve, PMA: phorbol 12-myristate 13-acetate.

### Production of ROS in CSF in cohort 1

**Fig 1:**
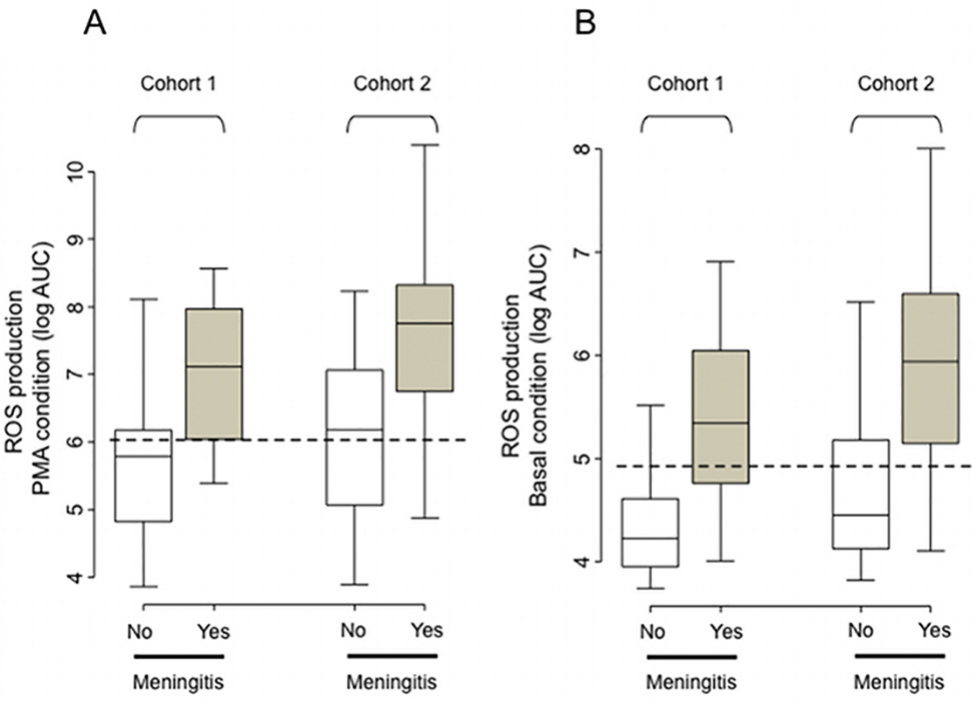
ROS production after PMA stimulation. ROS production after PMA stimulation (A) and in the basal state (B) in cohorts 1 and 2 according to an expert diagnosis of meningitis.

**Fig 2:**
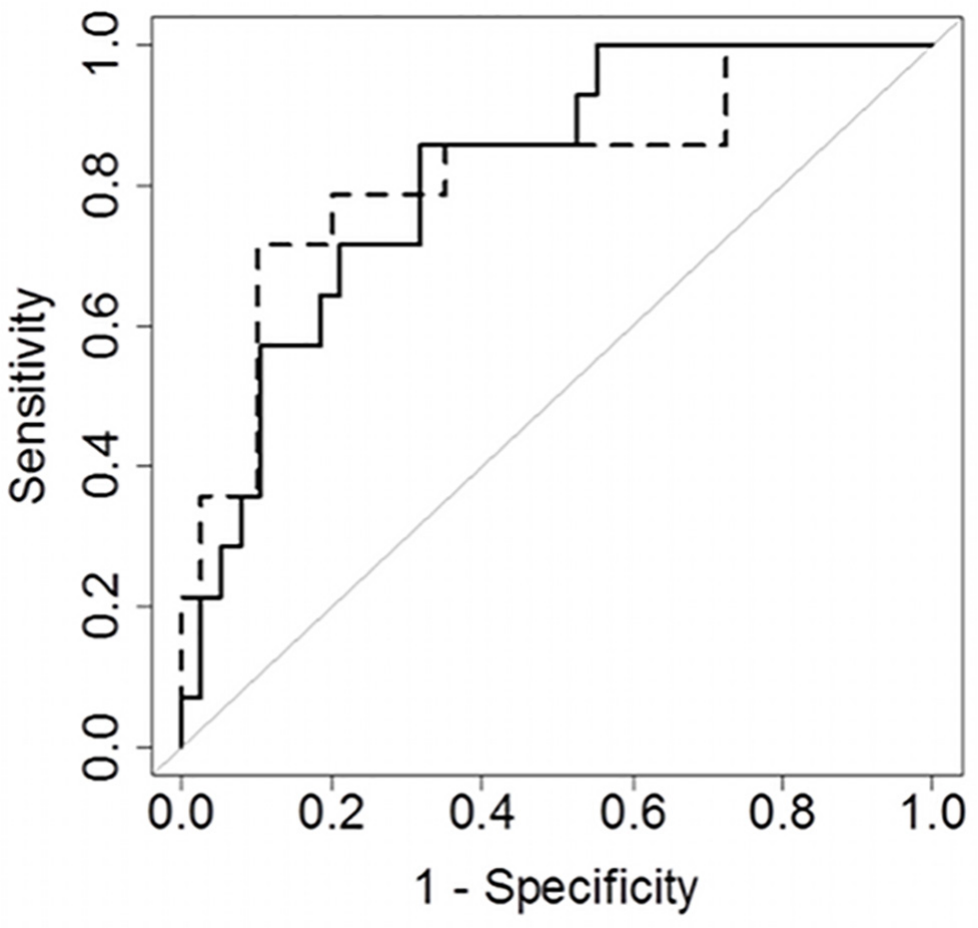
ROC curves associated with a diagnosis of meningitis. ROC curves associated with a diagnosis of meningitis in cohort 1 after PMA stimulation (solid line) and in the basal state (dotted line).

Among the parameters tested in cohort 1 (Table 2), the production of ROS was higher in CSF from patients with expert-diagnosed meningitis compared to patients with a negative diagnosis, both in the basal state and after PMA stimulation (Fig 1). ROS production in CSF was associated with an expert diagnosis of meningitis with an area under the ROC curve of 0.814 (95%CI [0.684–0.820]) in the basal condition and 0.818 (95% CI [0.655–0.821]) after PMA stimulation (Fig 2). The optimal cut-off for the basal condition was a luminescence AUC of 84900 with a sensitivity of 0.71 (95% CI [0.15–0.93]) and a specificity of 0.90 (95% CI [0.28–0.98]). The optimal cut-off for the PMA condition was a luminescence AUC of 1060673 with a sensitivity of 0.86 (95% CI [0.50–1.00]) and a specificity of 0.68 (95% CI [0.32–0.84]).

### Validation of the test in cohort 2

One hundred and thirty-six patients were enrolled in multicenter cohort 2. The clinical context, CSF characteristics and incidence of a diagnosis of meningitis are presented in Table 1. Meningitis was diagnosed in 21 patients in cohort 2. Twelve CSF samples were positive for *Staphylococcus*, enterobacteria, *Pseudomonas*, *Bacillus*, *Streptococcus pneumonia* and *Propionibacterium*. Discrepancies between the expert diagnosis and microbial findings in CSF were found in 11 patients (8.1%) (see supplemental digital content S1 Table).

Differences in clinical and biological parameters according to the diagnosis of meningitis in cohort 2 are detailed in Table 2. Differences in ROS production are shown in Fig 1. The association between ROS production in CSF and expert diagnosis of meningitis is represented by the areas under ROC curves, which were 0.812 (95% CI [0.680–0.815]) for the basal condition and 0.834 ((95% CI [0.723–0.836]) after PMA stimulation (see electronic supplement S1 Fig). The performance of the optimal cut-off of the test from cohort 1 was applied to cohort 2 in both conditions. In the basal state, the sensitivity of the test was 0.76 (95% CI [0.57–0.95]), the specificity was 0.67 (95% CI [0.50–0.97], the negative predictive value (NPV) was 0.94 and the positive predictive value (PPV) was 0.30. After PMA stimulation, the sensitivity of the test was 0.89 (95% CI [0.66–1.00]), the specificity was 0.47 (95% CI [0.14–0.75]), the NVP was 0.96 and the PPV was 0.23. The negative and positive likelihood ratios for a diagnosis of meningitis were 0.36 and 2.3, respectively, for the basal condition and 0.24 and 1.66, respectively, after PMA stimulation.

### Sensitivity analysis of the ROS test

**Fig 3:**
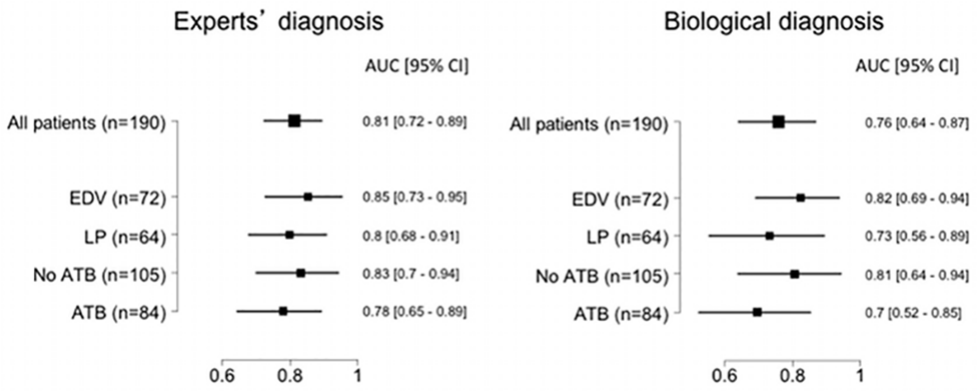
Performance of ROS measurements. The performance of ROS measurements (basal state) in the total cohort of 190 patients according to sampling by LP or EVD, the presence or absence of previous antibiotic administration and a diagnosis of meningitis by the experts or biological criteria (i.e., CSF cells > 100/mm^3^ and positive microbiological results [5]).

In the total population of 190 patients, the performance of the ROS test was compared to the diagnosis of meningitis made by the experts or with classical biological criteria (i.e., CSF cells > 100/mm^3^ and positive microbiological results [5]). The performance of the ROS test was similar to both expert and biological diagnoses, and in the basal state the AUCs were 0.81 [0.72–0.89] and 0.76 [0.64–0.87], respectively (Fig 3). After PMA stimulation the respective AUCs were 0.79 [0.70–0.88] and 0.76 [0.65–0.85] (S3 Fig).

Because the routes of sampling, lumbar puncture (LP) or external ventricular drainage (EVD), have different risk factors for meningitis or ventriculitis, we tested ROS production for both types of sampling. The performance of the ROS test was similar to that of expert diagnosis if samples were obtained by LP or by EVD in the basal state (Fig 3). However the ROS test appears to be more accurate in EVD than in LP samples compared with biological diagnosis (Fig 3). Conversely, the performance of the ROS test appears to be better in LP than in EDV samples after PMA stimulation (S3 Fig).

Because the concomitant administration of antibiotics for other infections could alter the course of meningitis, we analyzed the results of the ROS test in patient subgroups who were or were not taking antibiotics (Fig 3 and S3 Fig). The data lead to the suggestion that ROS production may be less affected by antibiotic therapy in the basal state than after PMA stimulation.

Additionally, ROS results were not influenced by age or gender.

### Performance of the ROS test for early identification of meningitis

Because early suspicion of meningitis is limited to biological criteria, we compared the performance of the ROS test with patients who had CSF cell counts >100/mm^3^ in the total population of 190 patients. The ROS test in the basal state and after PMA stimulation and high CSF cell counts (>100/mm^3^) had sensitivities of 0.74, 0.88 and 0.89, specificities of 0.73, 0.53 and 0.75, NPVs of 0.93, 0.95 and 0.97 and PPVs 0.38, 0.30 and 0.44, respectively.

## Discussion

This study aimed to validate the production of ROS in CSF as a marker of meningitis in one-center cohort first, then with a second multicenter cohort of febrile patients (over 38°C) in a context of TBI or neurosurgery. The proportion of meningitis diagnosed by experts was 25.9% in cohort 1 and 15.4% in cohort 2. In both cohorts, the production of ROS in CSF was higher in diagnosed meningitis compared with negative CSF under both measurements condition (basal and PMA stimulation). The test demonstrated high sensitivity and negative predictive value for prediction of CSF infection. This test may constitute a rapid and reliable tool for eliminating meningitis and particularly when it is suspected in a context where biological diagnosis is difficult, for example soon after intracranial hemorrhage or surgical procedures.

In neurocritical care, suspicion of meningitis is a daily concern when fever occurs after brain injury or neurosurgery. In this context, the presence of PMN in CSF may result from sterile inflammation or from a proven infection [5]. Even these criteria may be confusing because positive cultures may result from contamination [3] and negative cultures may result from growth that is limited by antibiotic treatment. Because of these difficulties and in the absence of a consensus on CSF criteria for use in diagnosis of potential cases of meningitis, we decided to use blinded-expert diagnosis as a reference for evaluating the test. All of the clinical and microbiological information was made available to the two trained experts in our study when they reviewed the cases, but results from the ROS test were not provided. They diagnosed a similar proportion of meningitis in the 2 cohorts (26% in cohort 1; 15% in cohort 2), an incidence that is in agreement with previous reports [3,5].

Looking at ROS production is a reasonable approach because the main source of ROS is activated PMNs, mainly via the NADPH oxidase pathway [6,10]. In addition to the number of PMNs in CSF, their degree of activation may also influence the intensity of ROS release [7]. It is then possible that the activation of PMNs reaches higher levels in the presence of bacteria compared with other stimuli. The observed production of ROS in our samples supports this hypothesis both in the basal state and after stimulation because the quantity of ROS correlated with the number of cells in the CSF (see electronic supplement, S2 Fig) both in infected and non-infected samples. The ability of PMNs to cross the blood-brain barrier, called PMN recruitment, results from complex mechanisms, among which ROS release predominates by facilitating PMN migration [11]. Increased expression of the enzyme NADPH oxidase in PMNs may also participate in higher ROS release in cases of infection [12].

A suspicion of meningitis is very frequent in neurocritical practice and is a daily concern, with a heterogeneous incidence ranging from 1 to 20% [1], depending on the clinical context and the likelihood of a higher incidence in a febrile population of patients, as highlighted in our study. In keeping with the principle of precaution, test sensitivity must be very high to allow reliable selection of negative cases before pursuing costly investigations and treatments. After application of the test to cohort 1 in a single center, we reproduced the results in a second multicenter cohort. This constitutes the first step of validation of ROS production as a marker of CSF infection. The high sensitivity and the high negative predictive value for meningitis allows the provider to eliminate a diagnosis of meningitis in a large population of patients with various infection exposure risks, such as trauma, postoperative cerebrospinal fluid leakage, and external drainage. The positive predictive value (PPV) is less important than negative predictive value (NPV) because investigations (CSF analysis) and treatments (antibiotics) are neither high-risk nor invasive procedures, and a final diagnosis of meningitis can be confirmed or invalidated later using complete biochemical and microbial results. The limited PPV of our test to diagnose meningitis may result from recruitment of PMNs to the CSF due to mechanisms independent of infection, such as tissue lesions, release of chemoattractive molecules from blood degradation products or surgical compounds used for hemostasis, such as thrombin, fibrin glue or cellulose polymer. These mechanisms have contributed to the failure of previous biomarkers of oxidative damage or anti-oxidative enzymes to discriminate sterile versus infected CSF inflammation [13,14]. Such oxidative products become stable and accumulate in CSF, making such measurements less accurate for the diagnosis of secondary infection. Conversely, our test was based on the instantaneous production of ROS, which clearly reflects the ongoing intensity of oxidation processes in the CSF. By nature, the radicals are unstable and require rapid measurements. We have demonstrated that our method provides reproducible measurements over several hours [7].

We described the sensitivity analysis of our parameters because the requirement to diagnose/exclude a diagnosis of meningitis occurs in different contexts and also because the interpretations of CSF parameters by physicians may vary in the absence of recommended definitions and thresholds for variables [8]. Our interest in ROS production was confirmed for diagnoses based on classical biological criteria, including CSF cell counts > 100/mm^3^ and positive microbiology [5]. ROS results could also be altered by different clinical conditions such as ventriculitis related to CSF drainage versus global meningitis after trauma and neurosurgery. We therefore examined the impact of the site of CSF sampling on the measurements with regard to to the ventricular site (EVD) at the origin of CSF production and the lumbar site (LP) in the dural sac. Our interest in ROS measurements was also confirmed in both LP and EVD samples, *i*.*e*., global CSF infection and ventriculitis. Finally, antibiotics were administered to 44% of our population for other infections, which could alter a diagnosis of central nervous system infection, but the ROS test still performed well and especially in comparison with the expert diagnoses. The clinical context and comorbidities like diabetes might have an impact on ROS release but were not tested in this study. Further data collection will help to clarify these important questions.

Other types of biomarkers, such as serum procalcitonin [15,16], a combination of CSF glucose, lactate and cell count [17], cytokines in CSF [18] and 16S ribosomal RNA in CSF [19], have been investigated for diagnosing infection in CSF, but none have improved the accuracy in comparison with classical criteria, and some were unsuitable for use with all of the patients studied in this hospital context. The results of our test were not different from those using classical criteria, but the test was well-adapted to allow quick results, bedside use, and to permit rapid selection of suspect CSF samples (before completing other investigations and beginning/changing treatment). Classically, a suspicion of meningitis is verified by CSF cell counts, glucose and protein levels, and then antibiotics are administered before microbiological results are available. This protocol requires time and qualified manpower, and a unique ROS test may simplify the procedure and help decision-making. For example, we compared two early strategies using the ROS test (basal and PMA conditions) and CSF cell counts alone. In this example, after PMA stimulation, the ROS test is as sensitive and has the same NPV as a CSF cell count higher than 100/mm^3^. ROS measurements would be superior to cell counts for diagnosing infection if, for the same number of cells, ROS production was higher in infected than in non-infected CSF. This hypothesis was supported by *in vitro* studies which have demonstrated increased NADPH oxidase subunit expression after stimulation of cells by LPS or interferon gamma [12]. As illustrated with the correlation between ROS production and cell count in CSF (S2 Fig), the straight line associated with infected samples was above the line associated with non-infected samples, suggesting higher ROS production in conditions of infection. However, the difference was not sufficiently marked to allow confirmation of this hypothesis without carrying out further studies. Such a difference was not observed when cells were stimulated by PMA, suggesting that a strong activation stimulus may mask such subtle differences.

The limitations of the current study include the lack of a consensus for postoperative or post-trauma meningitis diagnostic criteria [1]. This has motivated the investigators to develop a more reliable method for diagnosing bacterial meningitis. An expert-based diagnosis, although it is not validated in the literature, appears well-suited in this clinical context. First, the experts analyzed patient data separately and differences in the analysis were observed, a joint analysis of the data led to a consensual diagnosis. A second limitation was the reproducibility of ROS measurements as demonstrated by the differences in global luminescence levels between cohorts 1 and 2, despite the similar performance of the test in both cohorts. This may result from differences in the experimental conditions, used to study cohorts 1 and 2. Because we measured instantaneous ROS production by fresh cells, we could not use standard samples as a reference. A variation in luminol reactivity over time may occur, and development of a standardization protocol, including a protocol for determining reagent quality is therefore justified.

## Conclusion

This data obtained in this prospective multicenter study confirmed the interest studying ROS measurements in the diagnosis of meningitis. The performance of the test was excellent for its sensitivity and negative predictive value in comparison with diagnoses made by two independent experts. In order to validate the use of ROS measurements in the detection of CSF infection, the test requires further standardization and development for rapid bedside use. Such a test would help decision-making by clinicians and limit numerous specialized CSF lab tests and the administration of broad-spectrum antibiotics.

## Supporting Information

S1 Fig ROC curves associated with a diagnosis of meningitis. ROC curves associated with a diagnosis of meningitis in cohort 2 after PMA stimulation (solid line) and in the basal state (dotted line).
(TIFF)

S2 Fig Correlation between CSF cell counts and ROS production. Correlation between CSF cell counts and ROS production (AUC) in the total population according to the experimental condition (basal state or after PMA stimulation) and a diagnosis of meningitis (presence (red) or absence (blue) of infection).
(TIFF)

S3 Fig Performance of ROS measurements. The performance of ROS measurements (PMA-stimulated) in the total cohort of 190 patients according to sampling by LP or EVD and the presence or absence of previous antibiotic administration for a diagnosis of meningitis by the experts or biological criteria (i.e., CSF cells > 100/mm^3^ and positive microbiological results [5]).
(TIFF)

S1 Table Patients with discrepancies between adjudicated diagnosis of meningitis and microbiology. Individual information and values in patients with discrepancies between adjudicated diagnosis of meningitis and microbiology. Clinical context and biological data are reported.
(DOC)
