# Supplementary figures and images for: Multicenter Testing of the Rapid Quantification of Radical Oxygen Species in Cerebrospinal Fluid to Diagnose Bacterial Meningitis
Source: PLoS One. 2015 May 26;10(5):e0128286. doi: 10.1371/journal.pone.0128286 (PMC4444193; doi:10.1371/journal.pone.0128286)

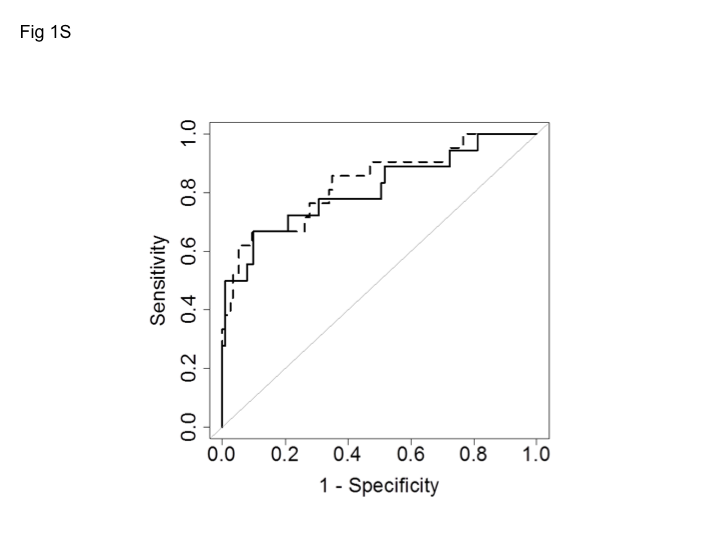

Supplement: S1 Fig — ROC curves associated with a diagnosis of meningitis in cohort 2 after PMA stimulation (solid line) and in the basal state (dotted line). (TIFF) [file pone.0128286.s001.tiff]

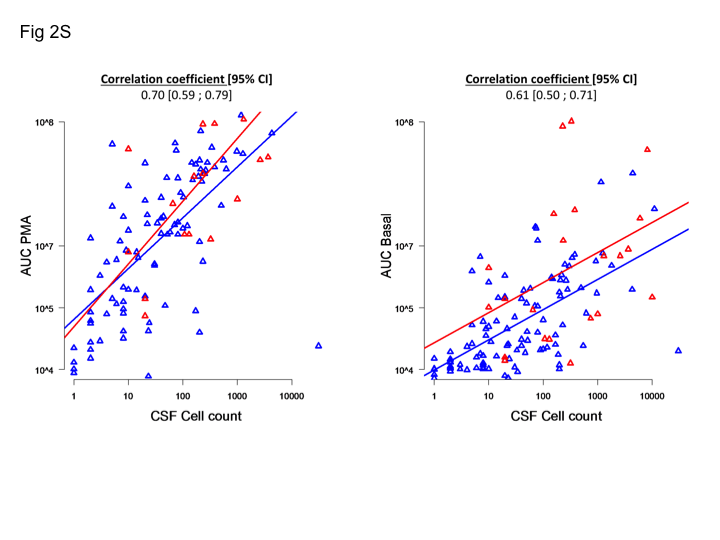

Supplement: S2 Fig — Correlation between CSF cell counts and ROS production (AUC) in the total population according to the experimental condition (basal state or after PMA stimulation) and a diagnosis of meningitis (presence (red) or absence (blue) of infection). (TIFF) [file pone.0128286.s002.tiff]

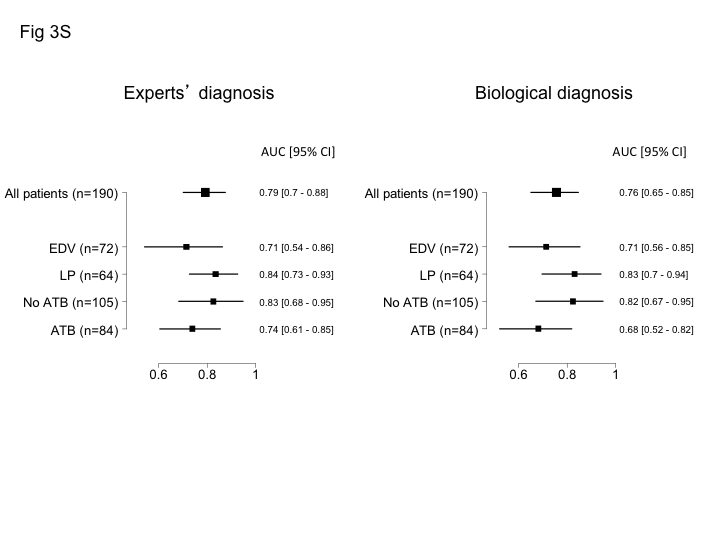

Supplement: S3 Fig — The performance of ROS measurements (PMA-stimulated) in the total cohort of 190 patients according to sampling by LP or EVD and the presence or absence of previous antibiotic administration for a diagnosis of meningitis by the experts or biological criteria (i.e., CSF cells > 100/mm3 and positive microbiological results [5]). (TIFF) [file pone.0128286.s003.tiff]
