# Supplementary material for: Multicenter Testing of the Rapid Quantification of Radical Oxygen Species in Cerebrospinal Fluid to Diagnose Bacterial Meningitis
Source: PLoS One. 2015 May 26;10(5):e0128286. doi: 10.1371/journal.pone.0128286 (PMC4444193; doi:10.1371/journal.pone.0128286)
Supplement: S1 Table — Individual information and values in patients with discrepancies between adjudicated diagnosis of meningitis and microbiology. Clinical context and biological data are reported. (DOC) [file pone.0128286.s004.doc]

Supplemental digital content:

Table S1: Individual information and values in patients with discrepancies between adjudicated diagnosis of meningitis and microbiology. Clinical context and biological data are reported.

| N° | cohort | Context | Temp (°C) | WBC (/mm^3^) | CSF cells  (/mm^3^) | % PMN in CSF | RBC in CSF  (/mm^3^) | Glucose in CSF  (mmol/L) | Proteins in CSF  (g/L) | Glycemia  (mmol/L) | W/R ratio in CSF | CSF/Blood glucose | Adjudicated diagnosis of meningitis | microbiology | ATB |
| --- | --- | --- | --- | --- | --- | --- | --- | --- | --- | --- | --- | --- | --- | --- | --- |
| 1 | 1 | Postop | 39.1 | 15.2 | 210 | 96 | 71000 | 2.3 | 3.2 | 5.1 | <1 | 0.45 | Pos | Neg | Y |
| 2 | 1 | EDV | 40.1 | 9.5 | 23000 | 95 | 200 | 3.5 | 3.1 | 16.2 | 11500 | 0.22 | Pos | Neg | N |
| 3 | 1 | Postop | 37.7 | 12.9 | 2100 | 87 | 5 | 2.2 | 3.1 | NA | 42000 | NA | Pos | Neg | N |
| 4 | 1 | TBI | 37.8 | 20.9 | 900 | 78 | 10900 | 2.4 | 22 | 7.5 | 8 | 0.32 | Pos | Neg | N |
| 5 | 1 | EDV | 38.7 | 9.2 | 572 | 73 | 37 | 2.6 | 1.3 | NA | 1546 | NA | Pos | Neg | N |
| 6 | 1 | EDV | 39.3 | 21.4 | 18 | 3 | 3700 | NA | NA | 10.2 | <1 | NA | Neg | Pos | Y |
| 7 | 1 | EVD | 39.6 | 9.6 | 30 | NA | 330000 | 5.9 | 0.5 | 8.7 | <1 | 0.68 | Neg | Pos | Y |
| 8 | 2 | Postop | 39.5 | 20.7 | 1800 | NA | 62000 | 1.3 | 3.3 | 8.2 | 3 | 0.16 | Pos | Neg | Y |
| 9 | 2 | Postop | 37.5 | 13.8 | 4400 | 86 | 500 | 2.7 | 4 | 9.7 | 880 | 0.28 | Pos | Neg | N |
| 10 | 2 | Postop | 40.4 | 11 | 11000 | 98 | 130000 | 4.6 | 0.72 | 10.1 | 8 | 0.46 | Pos | Neg | Y |
| 11 | 2 | Postop | 38 | 18 | 1160 | 80 | 230 | 0.2 | 0.58 | 8.8 | 504 | 0.02 | Pos | Neg | Y |
| 12 | 2 | Postop | 38.6 | 13 | 75 | 73 | 0 | 1 | 0.9 | 5.8 | NA | 0.17 | Pos | Neg | N |
| 13 | 2 | EDV | 39 | 12.1 | 10 | NA | 15600 | 2.6 | 1.16 | 10.1 | <1 | 0.26 | Neg | Pos | Y |
| 14 | 2 | EDV | 40.2 | 10.1 | 10 | NA | 140000 | 4.9 | 0.16 | 6 | <1 | 0.82 | Neg | Pos | N |
| 15 | 2 | EDV | 38.3 | 9.6 | 158 | 53 | 11 | 4.7 | 0.3 | NA | 1436 | NA | Neg | Pos | N |
| 16 | 2 | Postop | 38.8 | 23.6 | 65 | 51 | 20000 | 7.8 | 0.3 | 13.7 | <1 | 0.57 | Neg | Pos | N |
| 17 | 2 | TBI | 38.8 | 10 | 20 | 49 | 2300 | 4.9 | 0.5 | 8.8 | <1 | 0.56 | Neg | Pos | N |
| 18 | 2 | EDV | 38.7 | 10.1 | 130 | 68 | 124000 | 7.2 | 0 | 12.3 | <1 | 0.59 | Neg | Pos | Y |

Postop : postoperative, TBI : trauma brain injury, EVD : external ventricular drainage, WBC: white blood cells, CSF: cerebrospinal fluid, PMN: polymorphonuclear cells, RBC: red blood cells, W/R: white/red, ATB: ongoing antibiotics, Pos : positive, Neg :negative, NA : non applicable.
